# A Study of Epstein-Barr Virus BRLF1 Activity in a *Drosophila* Model System

**DOI:** 10.1100/2012/347597

**Published:** 2012-04-26

**Authors:** Amy Adamson, Dennis LaJeunesse

**Affiliations:** Department of Biology, University of North Carolina at Greensboro, Greensboro, NC 27402, USA

## Abstract

Epstein-Barr virus, a member of the herpesvirus family, infects a large majority of the human population and is associated with several diseases, including cancer. We have created *Drosophila* model systems to study the interactions between host cellular proteins and the Epstein-Barr virus (EBV) immediate-early genes BRLF1 and BZLF1. BRLF1 and BZLF1 function as transcription factors for viral transcription and are also potent modifiers of host cell activity. Here we have used our model systems to identify host cell genes whose proteins modulate BRLF1 and BZLF1 functions. Via our *GMR-R* model system, we have found that BRLF1 expression results in overproliferation of fly tissue, unlike BZLF1, and does so through the interaction with known tumor suppressor genes. Through an additional genetic screen, we have identified several *Drosophila* genes, with human homologs, that may offer further insights into the pathways that BRLF1 interacts with in order to promote EBV replication.

## 1. Introduction

Epstein-Barr virus is a human herpesvirus that infects a majority of the human population. In addition to being the causative agent of infectious mononucleosis, Epstein-Barr virus (EBV) is also associated with several different cancers. Such malignancies include Burkitt's lymphoma, Hodgkin's lymphoma, nasopharyngeal carcinoma, breast cancer, and gastric cancer [1].

EBV can exist in a productive (lytic) phase or dormant (latent) phase. The EBV genome encodes more than 85 genes, subsets of which are expressed during the latent phase or during the lytic phase (which is broken down further into immediate-early, early, and late genes). BRLF1 (R) and BZLF1 (Z) are essential transcriptional activators expressed during the lytic phase that activate transcription of the EBV early genes. R and Z also have important roles in modulating the intracellular environment. For instance, R has been shown to interact with and alter the functions of the transcriptional regulators CREB-binding protein (CBP), Rb, and MCAF1 [2–4]. The Ran-binding-protein M (RanBPM) has also been shown to directly bind to R and act as a coactivator of R-mediated transcription [5]. R has been shown to promote cell cycle progression by activating S phase in fibroblast and epithelial cell lines [6], and conversely to promote senescence in an epithelial cell line [7]. Recently, R has been shown to inhibit expression of IRF3 and IRF7, leading to a decrease in the induction of interferon-*β* [8]. All of these findings have been accomplished via cell culture studies.

In order to study viral protein function in a more comprehensive way, we have created *Drosophila* model systems for both R and Z. We previously examined Z protein activity in *Drosophila* and were able to investigate Z's function at both the molecular and genetic level [9]. We identified the *Drosophila* gene *shaven* as a potent modifier of Z activity in fly tissue [9]. The human homolog of *shaven*, Pax5, also interacted with Z in human cells and plays an important role in EBV biology [9].

There are several cellular pathways that are important for cell cycle regulation within *Drosophila*. These pathways contain numerous tumor suppressors that, when mutated, contribute to tissue overgrowth. These tumor suppressors generally fall into one of three classes: hyperplastic (mutations that cause increased cell proliferation with normal tissue structure), such as those in the *target of rapamycin* (*Tor)* or *insulin receptor* pathways, neoplastic (mutations that cause increased cell proliferation with abnormal tissue structure and cause invasiveness) such as those in the *discs large (dlg)* or *Rab5* pathways, and nonautonomous (the overgrowth of wild-type cells due to neighboring cells being mutant) such as those in the *hyperplastic discs* (*hyd)* pathway [10]. Most of these tumor suppressors have human homologs that function in the same manner as in *Drosophila* cells.

Here we have made use of *Drosophila's* powerful genetic system to investigate R function and to investigate the pathways by which R may cause aberrant cell division. Via our *GMR-R* model system, we found that R expression causes overproliferation in fly tissue, as it has done in human cell culture. Through genetic screens, we have identified several *Drosophila* genes that are important for this R-mediated phenotype. The genes identified confirm previous findings from human cell culture and offer insights into how R interacts with host cell proteins and pathways to promote EBV replication.

## 2. Materials and Methods

### 2.1. Fly Culture

Flies were maintained at 20°C in plastic vials on a medium of cornmeal, yeast, molasses, and agar with methyl 4-hydroxybenzoate added as a mold inhibitor. *w^1118^* was used as the wild-type line. Fly stocks for the genetic screens were purchased from the Bloomington stock center. Crosses were performed at 20°.

### 2.2. P-Element-Mediated Transformation

The BRLF1 cDNA was cloned into the pGMR vector. Germline transformations were performed using the standard P-element protocol [11]. Several *GMR-R* lines were isolated.

### 2.3. Scanning Electron Microscopy

Flies were stored in 95% ethanol until ready to be sputter-coated. Flies were dried briefly, mounted onto stubs, and sputter-coated with gold. Sputter-coated flies were imaged in a Leica scanning electron microscope and images recorded at 2000x and 500x magnifications.

### 2.4. Immunostaining of Imaginal Discs

Eye-antenna imaginal discs were immunostained as described [12]. The anti-R antibody (Argene) was used at a 1 : 50 dilution and the anti-phospho-histone H3 antibody (Upstate) used at a 1 : 1000 dilution. Each primary antibody was incubated with several (~10) discs overnight. The secondary antibodies (donkey anti-mouse CY3 and donkey anti-rabbit FITC (Jackson Immunoresearch)) were used at a 1 : 2000 dilution, and were incubated with the discs for 2 hr. Discs were mounted in anti-fade media (Dako Cytomation). Images were obtained by confocal microscopy and analyzed with FluoView software and MicroSuite software.

## 3. Results

### 3.1. BRLF1 Produces a Dose-Sensitive Rough Eye Phenotype in *Drosophila*

**Figure 1:**
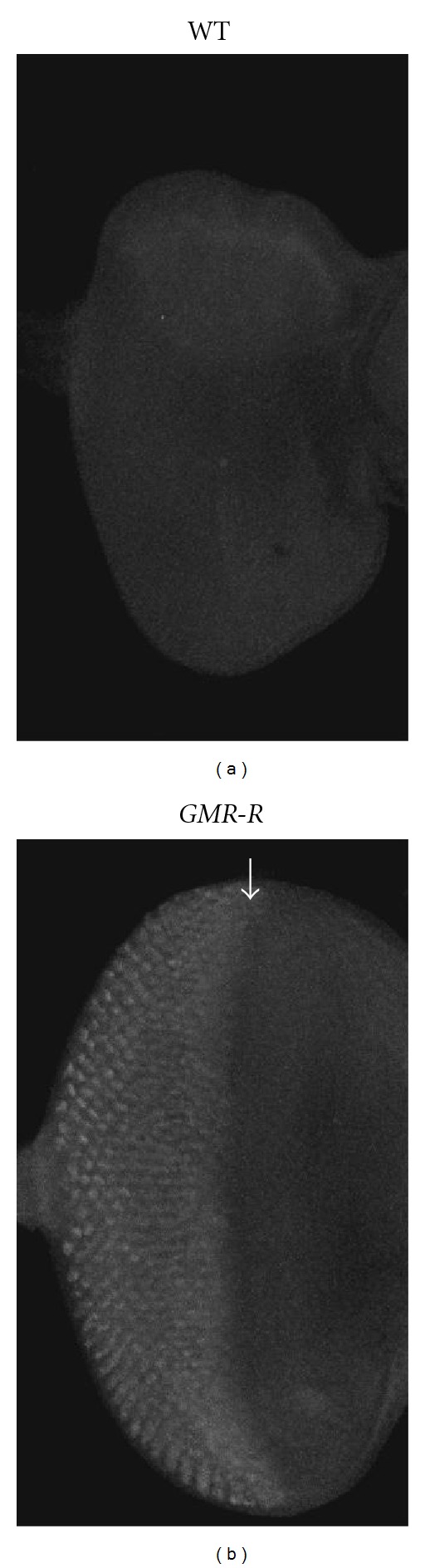
BRLF1 protein expression in the *GMR-R*/+ eye imaginal disc. Wild-type (a) and *GMR-R/+* (b) imaginal eye discs were stained with an anti-BRLF1 antibody. Confocal microscopy was used to image the discs. The arrow refers to the morphogenetic furrow.

**Figure 2:**
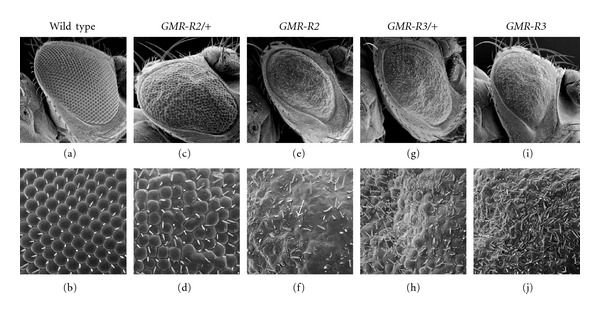
The *GMR-R* phenotype. SEMs of wild-type (a, b), *GMR-R2* heterozygous (c, d), *GMR-R2* homozygous (e, f), *GMR-R3* heterozygous (g, h), and *GMR-R3* homozygous (i, j) eyes are presented. The images are presented as 500x (top panels) and 2000x (bottom panels). Arrow points to hair-like bristles.

**Figure 3:**
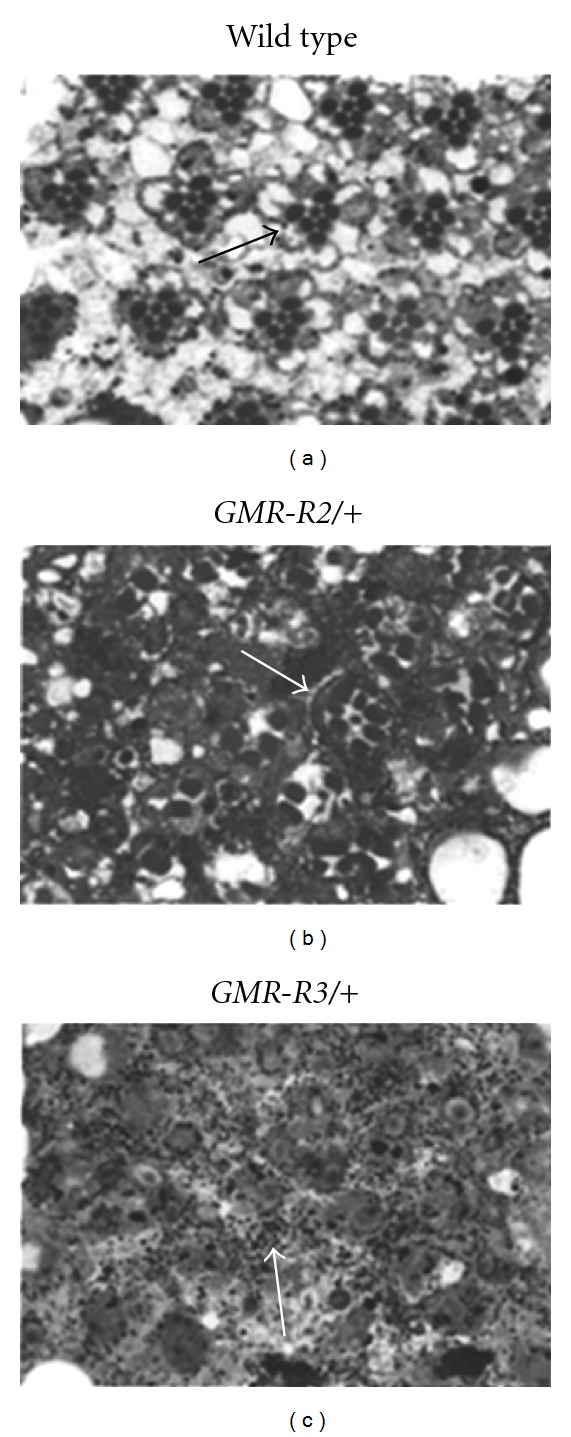
Sections of *GMR-R* adult eyes. Adult eyes from wild-type (a), *GMR-R2/+* (b) and *GMR-R3/+* (c) were embedded and sectioned. Arrows in (a) and (b) refer to photoreceptor clusters, arrow in (c) refers to pigment granules. Note that the photoreceptor cluster in (b) contains eight photoreceptors instead of seven.

BRLF1 transgenic flies were created by cloning the BRLF1 cDNA into the *Drosophila* P-element vector pGMR (Glass-mediated response) [13]. This vector allowed for eye-specific expression of BRLF1. Expression from this construct begins during the larval stage, with a peak during the third larval instar and can be seen in cells posterior to the morphogenetic furrow in eye imaginal discs (Figure 1(b)). Several lines of *GMR-R* were obtained, each with a dose-sensitive phenotype. Expression of BRLF1 in the *Drosophila* eye resulted in a rough adult eye phenotype (Figure 2). While wild-type eyes had an organized pattern of ommatidia (Figures 2(a) and 2(b)), flies heterozygous for *GMR-R2* (*GMR-R2/+*) had an unorganized ommatidial and bristle structure (Figures 2(c) and 2(d)). Flies homozygous for *GMR-R2* had a more severe eye phenotype, including a loss of ommatidia, and included the appearance of short hair-like bristles (Figures 2(e) and 2(f)). The *GMR-R* line *GMR-R3* (Figures 2(g)–2(j)) displayed a more severe phenotype than the *GMR-R2* line (Figure 2(c)–2(f)). The number of short, hair-like bristles was greatly increased, especially in homozygous *GMR-R3* eyes. *GMR-R* flies, and especially *GMR-R3* flies, had a darker red eye color than wild type (data not shown). When adult eyes were sectioned, we found that *GMR-R2/+*, with the more mild phenotype (Figure 2(d)), had disorganized but recognizable ommatidia (Figure 3(b)), while *GMR-R3/+*, with the more severe phenotype (Figure 2(h)), had no recognizable ommatidial structures, but had an overabundance of pigment granules (Figure 3(c)). 

### 3.2. Expression of BRLF1 Causes Overproliferation of Eye Cells

**Figure 4:**
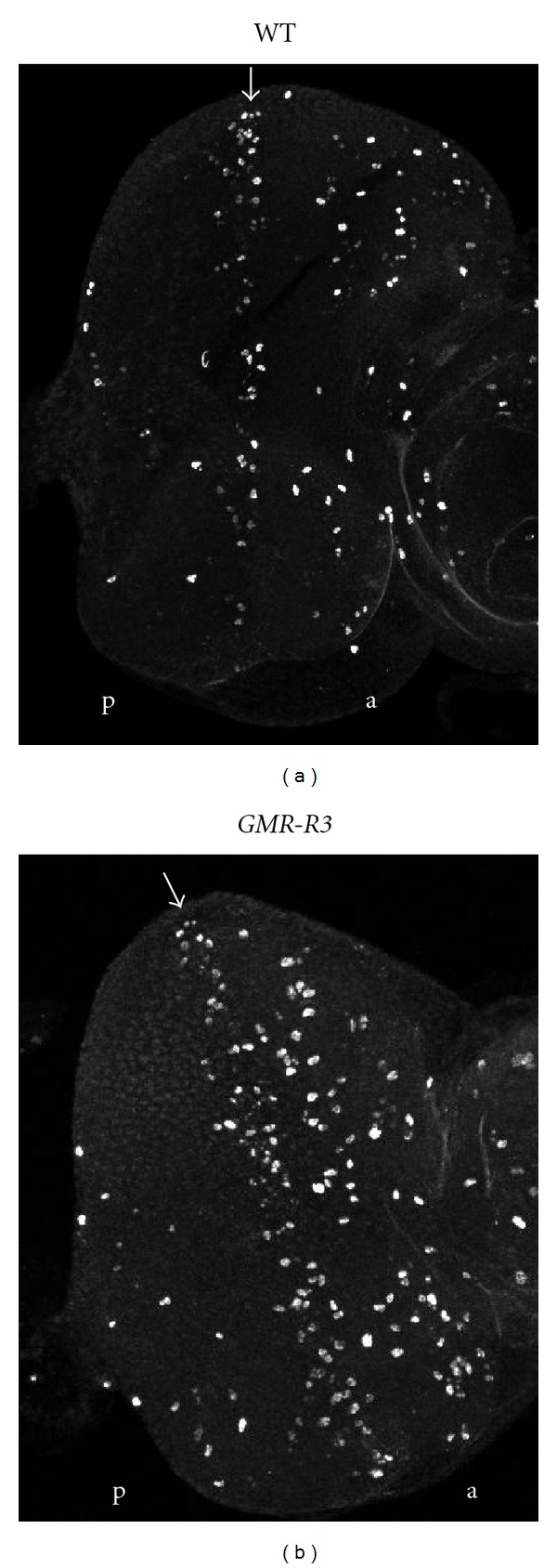
Increased cell division in *GMR-R* eyes. Third instar eye imaginal discs from wild-type (a) and *GMR-R3 *(b) larvae were stained with anti-phospho-histone-H3 (Ser 10) antibody. Confocal microscopy was used to image the discs. Arrows refer to the morphogenetic furrows. Posterior is to the left of each morphogenetic furrow.

**Figure 5:**
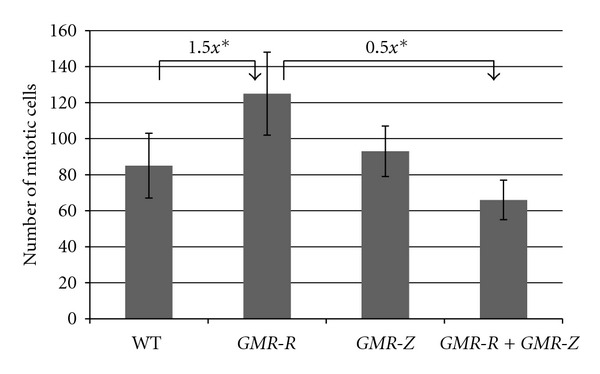
Expression of Z counteracts the overproliferation phenotype within *GMR-R* eye discs. Third instar eye imaginal discs from wild-type (WT), *GMR-R3, GMR-*Z,* and GMR-R/GMR-*Z larvae were stained with anti-phospho-histone-H3 (Ser 10) antibody. The number of positively stained cells within the furrow or posterior to the furrow was counted. Cells from approximately 12 discs were counted for each genotype. Error bars show the standard error; *indicates a *P* value < 0.0001.

As previous work suggested that BRLF1 expression increased entry into the cell cycle in tissue culture cells [6], we tested whether BRLF1 also increased entry into the cell cycle in *GMR-R* discs. To identify mitotic cells in the eye discs, we stained wild-type and homozygous *GMR-R3* discs with an anti-phospho-histone H3 (Ser 10) antibody. As histone H3 is phosphorylated on Ser 10 during mitosis, this antibody identifies mitotic cells only. In wild-type discs, mitotic cells were seen in the morphogenetic furrow, with some mitotic cells anterior to and posterior to the furrow (Figure 4(a)). In *GMR-R3* discs, more mitotic cells were found in the morphogenetic furrow as well as more mitotic cells posterior to the furrow (Figure 4(b)). The number of cells in the furrow and that posterior to the furrow were counted in wild-type and *GMR-R3* discs.  Figure 5 shows that *GMR-R3* discs have an average of 1.5 times more cells in mitosis than wild-type discs. Overproliferation was also evident in *GMR-R* adult eyes. Figure 3(b) shows a representative ommatidium that contained an extra photoreceptor cell. In addition, Figure 3(c) shows an overabundance of pigment granules, which contributed to the darker adult eye color in these flies.

### 3.3. Genetic Interaction between *GMR-R* and *GMR-*Z

We have previously analyzed the expression of the EBV BZLF1 gene in *Drosophila* [9]. The *GMR-*Z*/+* phenotype was different than that of *GMR-R* and included a rough eye phenotype and diminished pigment. Homozygosity for *GMR-*Z (referred to as “strong Z”) led to a loss of ommatidia (leaving a smooth eye) and a complete loss of pigment. In our studies we found that strong Z inhibited the cell cycle and prevented the differentiation of cone cells in homozygous *GMR-*Z eyes [9].

**Figure 6:**
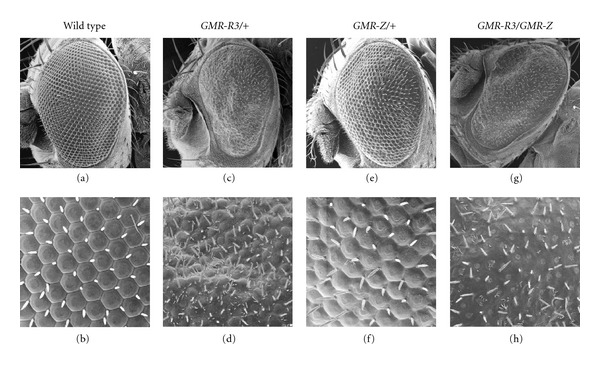
BZLF1 expression alters the *GMR-R* phenotype. A cross was performed between *GMR-R* and *GMR-*Z flies. SEMs were taken at 500x (top panels) and 2000x (bottom panels). Wild-type (a, b), *GMR-R3/+* (c, d), *GMR-*Z*/+* (e, f), and *GMR-R/GMR-*Z (g, h) adult eyes are presented.

Although the *GMR-R* and *GMR-*Z phenotypes seem to be antagonistic to each other (as R increases entry into the cell cycle and Z decreases entry into the cell cycle), during the normal EBV infection process, the two proteins R and Z are expressed at the same time and in fact work together to promote EBV gene expression [14]. Therefore we crossed *GMR-R3* and* GMR-*Z flies and examined their progeny (Figure 6). We also counted the number of mitotic cells in *GMR-R3/weak GMR-*Z larval eye discs. We found that while Z alone did not significantly alter the number of mitotic cells in eye discs, expression of Z did reverse *GMR-R3* overproliferation, such that the number of mitotic cells in *GMR-R3/weak GMR-*Z eye discs was half that of those in *GMR-R3* alone (resulting in a lower number than wild type) (Figure 5). This reversal of the *GMR-R3* phenotype can be seen in Figure 6(h), as the number of short hair-like bristles was also decreased. The eye color of the trans-heterozygotes was very similar to the *GMR-*Z*/+* eye color (orange), suggesting that the pigment cells had not over-proliferated as in *GMR-R3* eyes. However, Figures 6(g) and 6(h) shows that the *GMR-R3/weak GMR-*Z trans-heterozygous flies had a complete loss of ommatidia, although the bristles remained. This suggests that while Z counteracted the overproliferation phenotype of R, the presence of R and Z together elicited a significant cellular response causing the eventual loss of ommatidial cells.

### 3.4. *GMR-R* and *GMR-*Z Genetically Interact with Growth Regulator Mutants

**Figure 7:**
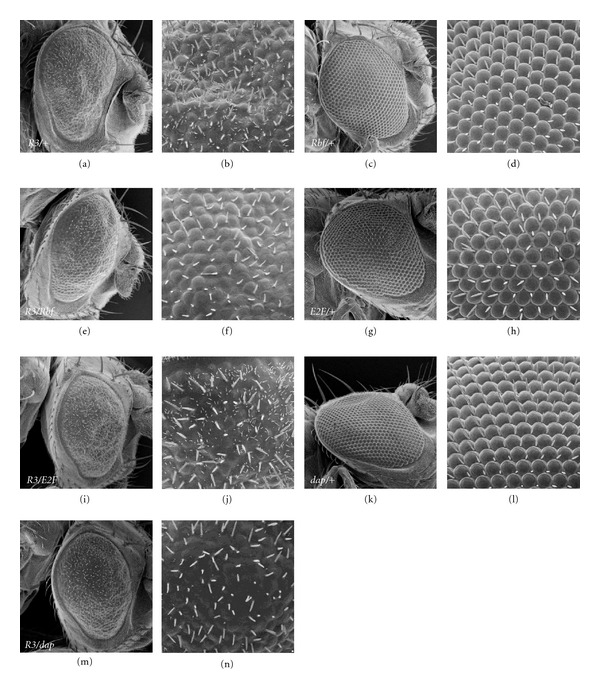
The *GMR-R* phenotype is suppressed by *Drosophila* Rb and p21 and enhanced by E2F. Crosses were performed between *GMR-R3* and *GMR-Rbf, GMR-E2F*, and *GMR-dacapo*. SEMs, at 500x and 2000x, are shown for *GMR-R3/+* (a, b), *GMR-Rbf/+* (c, d), *GMR-R3/GMR-Rbf* (e, f), *GMR-E2F/+* (g, h), *GMR-R3/GMR-E2F* (i, j), *GMR-dacapo/+ *(k, l), and *GMR-R3/GMR-dacapo* (m, n).

Both *R* and Z impact cell division, either positively or negatively. However, *GMR-R* and *GMR-*Z genetically interact to produce a more severe phenotype than either alone, suggesting that they participate in different pathways. To elucidate these pathways, we performed a genetic screen with candidate genes—known genes involved in growth regulation. We crossed both *GMR-R3* and *GMR-*Z to flies mutant for or overexpressing genes involved in cell cycle, signal transduction, and apoptosis. In most cases, more than one allele for each gene was tested. Enhancers of the *GMR-R* phenotype were those that increased the roughness/disorganization/pigmentation of the eye tissue, and/or increased the number of short hair-like bristles (e.g., see Figures 7(i) and 7(j)). Suppressors of the *GMR-R* phenotype were those that restored the eye to a more wild-type organization of ommatidia/pigmentation; this was typically accompanied by a reduction in the number of short hair-like bristles (e.g., see Figures 7(e), 7(f), 7(m), and 7(n)). Enhancers of the *GMR-*Z phenotype were those that led to a loss of ommatidia and pigmentation, while suppressors of the *GMR-*Z phenotype were those that restored wild-type ommatidial organization and pigmentation. Of the 51 genes tested, 15 modified the *GMR-R3* phenotype and 10 modified the *GMR-*Z phenotype (Table 1). Of these, there were 4 genes that modified both the *GMR-R* and *GMR-*Z phenotypes: *Rab5, p53, reaper*, and *Tor*.

Table 1 shows that fly lines that over-expressed the wild-type tumor suppressors *Rbf* (homolog of Rb), *dacapo* (homolog of p21), and *p53* suppressed the R overproliferation phenotype, while the overexpression of the cell cycle promoter *E2F* or a loss of a tumor suppressor (*brat, Csk, Merlin*) enhanced the R overproliferation phenotype. Conversely, fly lines that had a loss of a tumor suppressor (*hyd, l(2)gd1*) suppressed the Z phenotype, while the loss of cell cycle promoters (*Ras85*) enhanced the Z phenotype.

Interestingly, four fly gene mutants affected both the *GMR-R* and *GMR-*Z phenotypes. The *GMR-R* overproliferation phenotype was suppressed by over-expression of the cell cycle inhibitor *p53*, as well as by the proapoptotic gene *reaper*; the *GMR-*Z phenotype was enhanced by over-expression of both *p53* and *reaper*. Decreased levels of Tor suppressed the *GMR-R* phenotype, while the same mutants enhanced the *GMR-*Z phenotype. Furthermore, misexpression of *Rab5*, a GTPase that promotes endocytic vesicle fusion and helps terminate signaling pathways, enhanced both the *GMR-R* and *GMR-*Z phenotypes.

To further define R's biological role in cells, we performed a more objective genetic screen by crossing *GMR-R3* to the second chromosome EPgy2 misexpression line collection [15] (http://flystocks.bio.indiana.edu/Browse/in/misexpression-top.php). Each of these approximately 1000 lines tested contained a P element inserted either upstream of or within the coding region of a specific gene, causing that gene to either be overexpressed (if upstream) or mutant (if within). Lines that caused either enhancement or suppression of the *GMR-R* phenotype were crossed to a control line (*GMR* alone). Enhancers or suppressors that were specific to the *GMR-R* phenotype are presented in Table 2. Forty-nine genes were identified; only genes with human homologs and defined protein functions have been listed (22 of the 49). While some genes identified are involved in cell cycle regulation (*cdc14*), others are involved in signal transduction (*Paxillin, Rack1, 14-3-3 zeta, cdGAPr*) regulating the cytoskeleton (*RhoGEF2, genghis khan, Shroom, chickadee*) and vesicle transport (*beta coatomer protein, alpha-adaptin*) and specifically Rab GTPase activators (*CG16896, TRE oncoprotein related*). Others are transcriptional regulators (*defective proventriculus, bicoid interacting protein 3, TBPH*) or are involved in protein import into the nucleus (*Female sterile (2) ketel, PENdulin*). Interestingly, the overexpression of *RanBPM*, a scaffolding protein involved in many signal transduction pathways [16], suppressed the R phenotype; it has been previously reported that RanBPM physically interacts with R and promotes the transactivation ability of R in human B cells [5]. Also, while not listed in Table 2, the EP line screen identified *brat* as a potent modifier of the *GMR-R* phenotype, confirming our results in Table 1. *brat* does not have a human homolog, but functions as a tumor suppressor in *Drosophila* brain tissue [17].

## 4. Discussion

We have created a model system to investigate the cellular consequences of R expression. We found that R is expressed in the nuclei of eye cells while under the control of the GMR promoter element and that R expression causes more cells to enter the cell cycle. This overproliferation is evident from the *GMR-R* mutant eye phenotype—the ommatidia and bristles become unorganized, an overabundance of pigment leads to a dark red eye color, and short, fine bristles appear. These R-mediated effects are dose sensitive—the more R expressed, the more severe the phenotype. The coexpression of Z curbed the overproliferation phenotype in *GMR-R* eyes: fewer cells underwent mitosis in larval eye discs, and there was less pigment and fewer hair-like bristles in adult eyes. However the combination of R and Z did not produce a wild-type eye, as a different mutant phenotype took the place of both the *GMR-R* and *GMR-*Z phenotypes in *GMR-R/GMR-*Z eyes.

**Figure 8:**
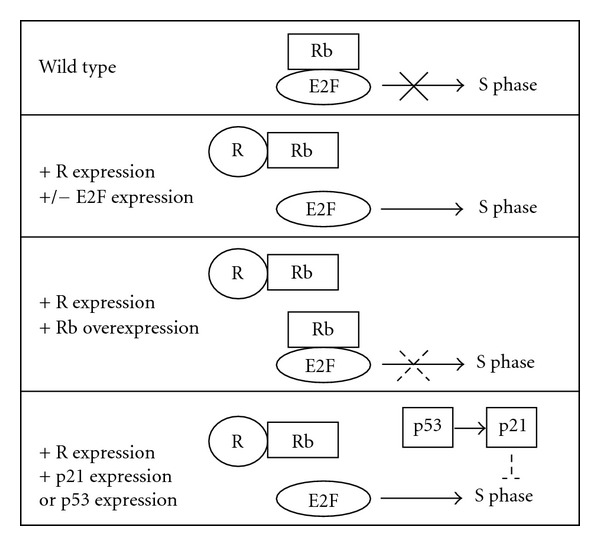
Model of R's effect upon S phase entry. Unless given a signal, cells will have Rb bound to E2F to inhibit E2F activity. R protein binds to Rb, displacing it from E2F, in which case E2F will promote S phase entry. Overexpression of E2F enhances this effect. Overexpression of Rb allows for renewed E2F inhibition. p53 or p21 overexpression inhibits cell cycle progression after it is initiated by active E2F.

In order to determine which cellular genes were mediating the *GMR-R* and *GMR-*Z phenotypes, we performed a candidate gene screen with both the *GMR-R* and *GMR-*Z flies, as well as an additional EP line screen with the *GMR-R* flies (Tables 1 and 2). Via the tumor suppressor candidate gene screen, we were able to determine that R's ability to promote the cell cycle is sensitive to the activities of a variety of cell cycle regulators, especially those falling into the *Drosophila* hyperplastic growth category. Our work confirms previous work showing that R interacts with Rb and E2F to promote entry into the cell cycle [3, 6] (for our model, see Figure 8), but also indicates that R activity is influenced by a number of signaling pathways, including the insulin receptor pathway and the Tor pathway. It is interesting to note that from this screen we found an equal number of enhancers for both R and Z (7 each), but more suppressors for R than for Z (8 for R versus 3 for Z). Therefore it appears to be “easier” to suppress R activity than to suppress Z activity.

From the EP line screen with *GMR-R*, we identified 5 suppressors and 17 enhancers of R activity. It is interesting to note that many of these modifiers code for proteins that are involved in protein/vesicle trafficking. Specifically, we identified two genes involved with protein transport into the nucleus: *Female sterile (2) ketel* and *Pendulin*. It appears that the likely overexpression of *Female sterile (2) ketel* and *Pendulin *leads to the enhancement of R activity, perhaps due to an increase of R protein import into the nucleus. Similar to our finding that *Rab5* was a modifier of both R and Z activity in our candidate gene screen, we identified other genes involved in vesicle trafficking, as well as Rab GTPase activators, as modifiers of R activity in this screen. Namely, the likely overexpression of *tre oncoprotein related* and *CG16896*, both Rab GTPase activators, enhanced the *GMR-R* phenotype, just as overexpression of *Rab5* (Table 1) enhanced the *GMR-R* phenotype. Rab proteins are involved in vesicle trafficking, and Rab5 is specifically involved in endocytosis [18].

Another class of genes that modify R activity are signal transduction mediators: *Paxillin, Rack1, RanBPM*, and *14-3-3zeta*. The fact that we found so many adaptors, which are involved in a variety of signal transduction pathways, to modulate R activity, indicates that the R protein exerts its effects via manipulation of several different signaling pathways.

We are very interested in the four genes that we identified in our candidate gene screen that modify both the *GMR-R* and *GMR-*Z phenotypes: *reaper, p53, Rab5*, and *Tor*. As both R and Z are present together in lytically replicating EBV-positive cells and they both contribute to transactivation of EBV early genes as well as to manipulation of their cellular environments, we are interested in cellular pathways that are common to both. We are especially interested in the mammalian mTOR pathway, which controls cell growth and protein translation [19] and how it will affect R and Z functioning during EBV lytic replication, as well as the mammalian Rab5 protein, and how vesicle trafficking may affect or be affected by EBV lytic replication.

Overall, we have established a model of R activity that mimics how the protein functions in human cells and have identified several cellular mediators of R activity that will be interesting foci for future study.

## Figures and Tables

**Table 1:** Candidate gene screen of tumor suppressors.

Allele	Nature of allele*	Effect
*14-3-3zeta^P1375^*	P element insertion	Enhancer of R
*brat^1^, brat ^k06028^*	EMS, P element insertion	Enhancer of R
*Csk^c04256^*	P element insertion	Enhancer of R
*GMR-E2F*	Overexpresser	Enhancer of R
*InR^E19^*	EMS	Enhancer of R
*Merlin^4^*	loss of function	Enhancer of R
*Rab5^EY10619^*	P element enhancer upstream,	Enhancer of R
*Rab5^KG05684^*	P element insertion	Enhancer of Z (*Rab5^EY10619^* only)
*ago^EY19092^*	P element insertion	Enhancer of Z
*dlg1^G0276^*	P element insertion	Enhancer of Z
*GMR-p53*	Overexpresser	Enhancer of Z
		Suppressor of R
*GMR-reaper*	Overexpresser	Enhancer of Z
		Suppressor of R
*Ras85^e1B^*	EMS missense mutation	Enhancer of Z
*Tor^DeltaP^, Tor^k17004^*	Deletion, P element insertion	Enhancer of Z
		Suppressor of R
*awd^j2A4^*	P element insertion	Suppressor of R
*chico^KG00336^*	P element insertion	Suppressor of R
*GMR-dacapo*	Overexpresser	Suppressor of R
*GMR-Rbf*	Overexpresser	Suppressor of R
*scrib^j7b3^*	Hypomorph	Suppressor of R
*hyd^15^*	EMS	Suppressor of Z
*l(2)gd1^EY04750^*	P element insertion	Suppressor of *Z *
*14-3-3epsilon^s-969^*	antimorph	Suppressor of Z

*Information about alleles from FlyBase.

EMS: ethyl methanesulfonate; hypomorph: less protein activity; antimorph: dominant negative protein activity.

**Table 2:** EP Line (second chromosome) Genetic Screen: Suppressors (S) and Enhancers (E) of the *GMR-R* phenotype.

Class	*Drosophila* gene	Insertion**	Human homolog	Function
S	Beta coatomer protein	Upstream	COPB2	Vesicle transport from Golgi
S	Defective proventriculus	Within	SATB1	Transcription/chromatin organization
S	Paxillin	Within	TGF*β*1I1	Signal transduction/adaptor
S	Rack1	Within	GNB2L1	Signal transduction
S	RanBPM	Upstream	RANBP9	Scaffolding/proapoptotic
E*	cdc14	Within/up	CDC14A	Phosphatase/cell cycle/p53
E*	Chip	Upstream	LDB2	LIM domain binding
E*	Female sterile (2) ketel	Upstream	KPNB1	Protein import into nucleus
E*	NAT1	Within/up	EIF4G2	Repressor of translation initiation
E*	Tre oncoprotein related	Upstream	TBC1D3C	Rab GTPase activator
E*	Yippee interacting protein 2	Within/up	ACAA2	Acetyl Coa-acyltransferase
E	14-3-3 zeta	Within	YWHAZ	Signal transduction/insulin pathway
E	Alpha-adaptin	Upstream	AP2A2	Clathrin-coated vesicle transport
E	Bicoid-interacting protein 3	Upstream	MEPCE	Transcription
E	cdGAPr	Within/up	AC108065	GTPase activator
E	CG16896	Upstream	WDR67	Rab GTPase activator
E	Chickadee	Within/up	PFN4	Cytokinesis
E	Genghis khan	Upstream	CDC42BPA	Phosphorylation/actin polymerization
E	Pendulin	Within/up	KPNA	Importin alpha 2/nuclear import
E	RhoGEF2	Within	ARHGF12	Rho GEF/actin dynamics
E	Shroom	Within	SHROOM3	Actin binding
E	TBPH	Upstream	TARDBP	Mrna binding/repressor of transcription

*Indicates a strong enhancer of the *GMR-R* phenotype.

**Indicates whether the p element insertion, containing the enhancer, lies upstream, where it likely causes overexpression of the gene, or within, where it likely interrupts the coding region. Within/up indicates that the p element lies in the 5′ untranslated region of the gene.
